# Three-Dimensional Printed Polylactic Acid (PLA) Surgical Retractors with Sonochemically Immobilized Silver Nanoparticles: The Next Generation of Low-Cost Antimicrobial Surgery Equipment

**DOI:** 10.3390/nano10050985

**Published:** 2020-05-21

**Authors:** Lazaros Tzounis, Petros I. Bangeas, Aristomenis Exadaktylos, Markos Petousis, Nectarios Vidakis

**Affiliations:** 1 Composite and Smart Materials Laboratory (CSML), Department of Materials Science & Engineering, University of Ioannina, GR-45110 Ioannina, Greece; 2 Department of emergency medicine, INSELSPITAL, Universitätsspital Bern, 18, 3010 Bern, Switzerland; pbangeas@gmail.com (P.I.B.); Aristomenis.Exadaktylos@insel.ch (A.E.); 3 Mechanical Engineering Department, Hellenic Mediterranean University, Estavromenos, 71004 Heraklion, Crete, Greece; markospetousis@hmu.gr

**Keywords:** fused deposition modelling (FDM), 3D printing, sonochemical deposition, silver (Ag) nanoparticles (NPs), antimicrobial (AM) properties, polylactic acid (PLA), additive manufacturing, rapid prototyping, surgical equipment

## Abstract

A versatile method is reported for the manufacturing of antimicrobial (AM) surgery equipment utilising fused deposition modelling (FDM), three-dimensional (3D) printing and sonochemistry thin-film deposition technology. A surgical retractor was replicated from a commercial polylactic acid (PLA) thermoplastic filament, while a thin layer of silver (Ag) nanoparticles (NPs) was developed via a simple and scalable sonochemical deposition method. The PLA retractor covered with Ag NPs (PLA@Ag) exhibited vigorous AM properties examined by a reduction in Staphylococcus aureus (*S. aureus*), Pseudomonas aeruginosa (*P. aeruginosa*) and Escherichia coli (*E. coli*) bacteria viability (%) experiments at 30, 60 and 120 min duration of contact (*p* < 0.05). Scanning electron microscopy (SEM) showed the surface morphology of bare PLA and PLA@Ag retractor, revealing a homogeneous and full surface coverage of Ag NPs. X-Ray diffraction (XRD) analysis indicated the crystallinity of Ag nanocoating. Ultraviolent-visible (UV-vis) spectroscopy and transmission electron microscopy (TEM) highlighted the AgNP plasmonic optical responses and average particle size of 31.08 ± 6.68 nm. TEM images of the PLA@Ag crossection demonstrated the thickness of the deposited Ag nanolayer, as well as an observed tendency of AgNPs to penetrate though the outer surface of PLA. The combination of 3D printing and sonochemistry technology could open new avenues in the manufacturing of low-cost and on-demand antimicrobial surgery equipment.

## 1. Introduction

The rapid prototyping has dramatically expanded over the last 20 y, with the three-dimensional (3D) printing in the forefront as one of the most promising technologies, amongst others [1,2]. 3D printing is an additive manufacturing process based on the sequential addition of material layers offering the opportunity to print 3D parts and components made of different materials with variable mechanical and physicochemical properties [3,4]. The first 3D print was reported by Hideo Kodama in 1982 [5]. Since then, the additive manufacturing of simple shapes, and especially with fused deposition modelling (FDM) technology in case of polymeric materials, has become much more pronounced. Nowadays, 3D printers are able to print with a multitude of materials, including metals, wood products, photopolymerizable resins, and thermoplastics such as polylactic acid (PLA). In addition, there are various techniques for printing solid materials in 3D, including electron beam freeform fabrication, direct metal laser sintering, and fused deposition modeling (FDM), among others [5].

3D printing is a significant technological attainment that could be deployed for a variety of applications in the medical field, overcoming existing limitations and providing significant improvements to state-of-the art technologies [6]. Recently, additive manufacturing has been intensely investigated for surgical equipment [5,7], implants [8], 3D bioelectronics [9], tissue scaffolds [10] and organs [11]. To date, the most important application of 3D printing in the medical field is the design and development of medical devices and instrumentation [12,13]. Additionally, surgeons have shown patient-specific, computed-tomography-derived 3D prints for better preoperative planning supporting the strategy of the surgical approach, specifically in complex operations [14,15] [16] and/or for educational purposes for young surgeons [17,18]. 3D models of patient-specific anatomy such as dental crowns and biological scaffolds are already being used for human implants [19,20,21]. There is, however, scant literature to date for the production of surgical instruments and equipment by 3D printing with some additional functionality resulting in multi-functional 3D objects, i.e., electrical conductivity, as a means to produce antistatic or self-heatable equipment (via Joule-heating effect), enhanced mechanical properties, sensing, anti-adhesive and antimicrobial properties to avoid biofilm formation with potential infections after the surgery, etc. [22].

Within the surgical world, Poly-Lactic Acid (PLA) and polyglycolic acids have gained a peculiar attention and have been intensely investigated for implants due to their thermoplastic nature, biodegradability, biocompatibility, mechanical strength and ease of processing [23]. As such, PLA could be a suitable material for printing surgical instruments. Moreover, another important characteristic of PLA is that it has the ability to be sterilized due to its relatively high melting point (typical Tm of PLA ~150–160 °C), which is necessary to its application for surgical equipment. Considering a surgery instrument, it should be sterile, and in the ideal case exhibit resistance to biofilm formation. This is because surgery that involves an incision in the skin can lead to a wound infection after surgery, while surgical wound infections may be painful or hot to touch. Specifically, PLA could be stable upon exposure to steam sterilization, however, it cannot withstand typical temperatures in the range of 160–170 °C for dry heat sterilization at 170 °C [21]. Other lower temperature sterilization methods, i.e., ethylene oxide “gas” sterilization did not impact PLA strength, although harmful levels of ethylene oxide residue are a serious concern. Alternatively, glutaraldehyde, which is known as an effective sterilant at room temperature, has been shown to retain the greatest PLA strength when compared with other chemical solution sterilants [24]. Moreover, minimal degradation of PLA has been reported for in vitro experiments, when physiological conditions are simulated for days to weeks [25]. 

**Figure 1:**
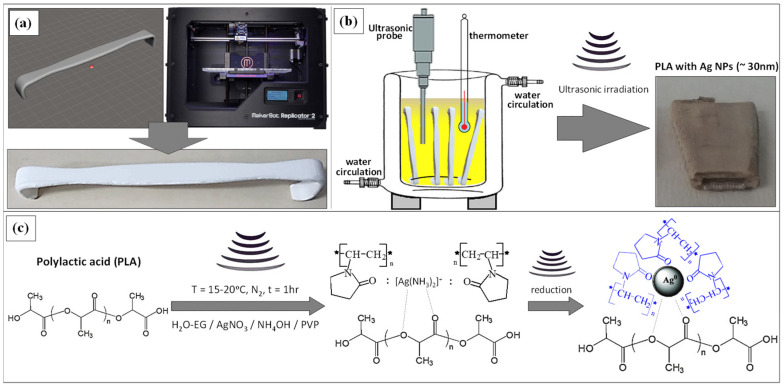
(**a**) 3D retractor model, 3D printer and 3D real object fabricated by fused deposition modelling (FDM) technology, (**b**) schematic illustration of the jacketed vessel reactor and all the equipment used for the sonochemical reaction and AgNP growth onto the polylactic acid (PLA) retractor surface (change in retractor color after the immobilization of AgNPs), and (**c**) analytic illustration of the reaction mechanism taken place to realize the growth and in-situ deposition of AgNPs to the PLA surface.

In recent years, a lot of attention has been paid to achieve planar surfaces of bulk materials, textiles, etc., with antimicrobial (AM) properties, especially in the health and hygienic field [26]. For this purpose, polymeric materials have been modified by incorporating antimicrobial agents in their bulk structure, i.e., by (i) solvent or (ii) melt-mixing. Another approach endowing AM properties in polymeric 3D objects is via depositing thin AM films onto their surface through wet or vacuum deposition technologies, while utilising specific interactions with polymer surface chemistry. Various AM agents have been extensively studied and results indicate the capability of preventing the growth of pathogenic microorganisms, such as bacteria, fungi, and algae. [27,28,29]. For instance, silver, copper, metal salts, quaternary ammonium compounds, polyhexamethylene biguanides and triclosan biopolymer chitosan are some of the AM agents that could be introduced in a polymer matrix or can be deposited as thin films on polymeric material surfaces [30,31]. Among the above-mentioned antibacterial agents, silver nanoparticles (AgNPs) have emerged as a new generation of antibacterial agents with a broad spectrum of antibacterial activity and low toxicity towards mammalian cells [32]. Although the silver antimicrobial mechanism is not fully understood to date, the most plausible mechanism could be related to specific interactions between AgNPs and the microorganism’s surface that promotes the silver penetration through the cell walls and reacts with the thiol group of proteins which results in cell death [33]. More specifically, Ag in its ionized form is highly active, as it binds to tissue proteins and brings structural changes to the bacterial cell wall and nuclear membrane, leading to cell distortion and death [34]. Unfortunately, the direct interaction of AgNPs with human cells inevitably leads to cytotoxicity and genotoxicity [35]. Therefore, it is crucial to immobilize and strongly bond the AgNPs onto the substrate material. In this way, the release of Ag NPs can be completely avoided and only a controlled release of Ag ions can be achieved. This immobilization/fixation approach has been intensively studied since it can suppress the potential hazardous influence of the AgNPs, while endowing the desired antibacterial properties [36]. The incorporation of silver in a polymer matrix by solvent mixing could be found elsewhere [37]. The deposition of silver onto the surface of various substrate materials has been reported using various deposition methods, i.e., magnetron sputtering [38], ion-beam-assisted deposition process [39], dip-coating using silver dispersions [40] and sonochemistry [41,42]. From the wet chemical deposition processes, the sonochemical method is very promising due to the versatility as well as the possibility of stabilizing the NPs onto the polymer substrate surface. This is more precisely explained by the following mechanism: the polymeric chains are partially swollen during sonication in case there is a strong or even weak polymer–solvent interaction, thus allowing thus the metal source ions, e.g., Ag^+^ towards AgNPs, Zn^+^ towards ZnO NPs, to penetrate into the polymer chains getting entrapped and further reduced during sonochemistry. This effect may contribute to a possible strong adhesion between the polymer surfaces with the deposited nanoparticles. The sonochemical method for thin film deposition onto polymeric surfaces has been widely used for different precursors, while that of immobilizing AgNPs is of great interest due to the unique AM properties of Ag, as stated above. However, it has not been implemented towards PLA surface modification, and specifically on a PLA consisting of a 3D-printed surgical instrument. Moreover, the advantages of our method lie in the fact that (i) monodispersed AgNPs have been formed onto the PLA surface following a versatile, straightforward, scalable and environmentally friendly synthetic protocol developed herein, (ii) chemicals, i.e., Ag precursor and capping/shape promoter agent compounds have been selected to allow for interaction of the Ag^+^ with the PLA macromolecular chains. As the AgNPs are reduced then, due to the ultrasound energy and grown in-situ onto the PLA surface (chemical interaction and growth mechanism are depicted in Figure 1c; AgNP layer morphology onto the PLA surface is shown by the SEM analysis in Figure 5d), and in (iii) it is envisaged that AgNP sonochemistry growth and wet-chemical deposition/immobilization onto the PLA surface can be scaled-up to the large-scale production of antimicrobial 3D-printed bulk surgery equipment without the need for advanced colloidal chemistry protocols for the synthesis of AgNPs and/or vacuum deposition techniques for the development of Ag nanolayers on 3D objects.

Herein, a surgical antimicrobial Army-Navy retractor, simple in shape and ubiquitous in all surgical specialties, has been fabricated using a versatile and scalable two-step process. Since PLA has been proven to be safe for surgical implantation, cost-effective, safe, and an environmentally suitable material for printing, a retractor was initially printed using a 3D-printing FDM rapid prototyping technology. AgNPs have been deposited then through a wet-chemical sonochemical deposition protocol, yielding extremely efficient retractor AM surfaces determined by the reduction in Staphylococcus aureus (*S. aureus*), Pseudomonas aeruginosa (*P. aeruginosa*) and Escherichia coli (*E. coli*) bacteria viability (%) after being exposed for 30, 60 and 120 min. Various characterisation techniques have been used in order to investigate the AgNP size as well as the film thickness and film homogenity over the PLA surface, demonstrating excellent film characteristics. It is envisaged that the proposed AM retractor could possibly substitute its standard stainless steel Army-Navy retractor counterpart, with an estimated cost of at least ten times lower.

## 2. Materials and Methods 

For the printing of a prototype replica of a common Army-Navy retractor, initially, a representative retractor model was designed in this work using a commercial 3D design Autodesk^®^ Fusion 360™ (Autodesk^®^, Inc., San Rafael, CA, USA) software, which was exported further to a Standard Tessellation Language (STL) file (Figure 1a). The MakerBot Replicator 2 FDM printer (MakerBot, Brooklyn, NY, USA) shown also in Figure 1a and the MakerBot MakerWare software that processes STL files into thin slices to generate G-code by means of slicing via MakerBot Slicer have been used (software products of MakerBot industries). The 3D object was printed with 75% infill (the density with which the instrument is printed), six shells of perimeter laid axially, and 0.20 mm layer height with a hexagonal infill pattern. The printer extruded PLA filament material (filament diameter: 1.75 mm) through a nozzle of 0.4 mm at 240 °C with a speed of 90 mm/s, while the bed temperature was set to 60 °C. The printing process required roughly 90 min. Each instrument weighed 16 g and required only 0.34€ of PLA.

For the deposition of AgNPs onto the surface of PLA retractor through a sonochemical deposition method, a printed retractor was placed initially in a cylindrical glass jacked vessel capable of being cooled down via water circulation and avoid overheating of the reaction medium by the ultrasonic irradiation (Figure 1b). For the sonochemical protocol, the optimum recipe previously reported in terms of AgNP size, antimicrobial activity, and coating homogeneity, has been followed with slight modifications [41]. In brief, a 250 mL solution of AgNO_3_ (0.02M) precursor has been prepared in a H_2_O/Ethyleneglycol (EG) medium (10:1 v/v) with EG acting as a polyol reducing agent An appropriate amount of Polyvinylpyrrolidone (PVP; average Mw ~10,000 g/mol) was added at a total concentration of 30 mg/mL, while the solution was transferred to the jacketed vessel and the whole system was purged with N_2_ for 1 h to remove traces of O_2_/air. In the next step, the solution was irradiated for 1 h with a high-intensity tip sonicator (Ti horn, 20 kHz, 600 W at 70% efficiency) under a N_2_ flow, keeping the reaction temperature constant at 15–20 °C. A 25% aqueous solution of ammonia (molar ratio NH_3_:AgNO_3_ = 2:1) was added to the reaction during the first 10 min of sonication. The addition of ammonia forms the [Ag(NH_3_)_2_]^+^ complex, while EG reduces the silver complex ion to metallic silver via the ultrasonic energy supplied to the reaction medium. The PVP introduced interacts with the Ag complex as shown in Figure 1c, and acts as the stabilizer and shape promoter agent to realize spherical NPs upon the reduction in the Ag complex to metallic Ag. PVP is also responsible for the AgNP-controlled size, resulting in uniform AgNP during growth, avoiding aggregation phenomena that can result in bad quality AgNPs. It should be mentioned that the equilibrium constant for the formation of [Ag(NH_3_)_2_]^+^ is large ≈ 10^7^, which dictates a small amount of Ag^+1^ in equilibrium with the complex. Therefore, the reduction process reaction kinetics is slowed down, enabling the creation of many Ag seeds that eventually lead to the formation of large and relatively monodispersed AgNPs during the sonochemical reaction. The overall reaction mechanism described above could be schematically shown in Figure 1c. At the end of the reaction, the color of PLA changed from white to gray (Figure 1b). Finally, the PLA@Ag was first washed thoroughly with water to remove traces of ammonia, then with ethanol, and dried overnight under vacuum. All the chemical reagents were purchased from Sigma Aldrich, were ACS grade and used as received without further purification. Figure 1 schematically shows all the steps followed to fabricate PLA@Ag antimicrobial retractors in this research work.

UV-vis spectra were recorded using a Cary 50 scanning spectrophotometer (Varian, USA) with an incorporated xenon flash lamp by using 1-cm quartz cell. X-ray diffractometry (XRD) was performed with an X-ray diffractometer (XRD Bruker D8 ADVANCE) in symmetric step-scan mode with Δ2Θ = 0.05° in transmission mode. The diffractometer operated at 40 kV and 30 mA with Cu Kα radiation (λ = 1.5406 Å), diffraction angle (θ, 10° < 2θ < 80°), and a step size of 5° at room temperature. Scanning electron microscopy (SEM) and the corresponding electron dispersive X-Ray spectroscopy (EDX) investigations were performed using the NEON 40 (Carl Zeiss AG, Oberkochen, Germany) scanning electron microscope under an accelerating voltage of 1.0 kV. Prior to the SEM analysis, a thin layer (3 nm) of platinum was deposited by sputtering onto the PLA and PLA@Ag retractor samples to avoid charging effects. Transmission electron microscopy (TEM) was carried out using a FEI transmission electron microscope operating at an acceleration voltage of 100 kV. All bright field TEM images were recorded inserting the appropriate energy filtering and contrast apertures in order to enhance the contrast of the images. Samples for TEM were prepared by dispensing 10 μL of free AgNPs from the solution after the end of the sonochemical reaction onto a Cu grid with a carbon support membrane. For the PLA@Ag crossection TEM imaging, a small part of PLA@Ag was cut in a rectangular shape and embedded in a room temperature 24 h curing epoxy resin. Afterwards, ultrathin sections of approximately 60–80 nm thickness were prepared by ultramicrotomy using a Leica ultramicrotome (Leica UC7, Leica Microsystems GmbH, Wetzlar, Germany) at room temperature, and more details can be found elsewhere [43,44,45,46]. Diamond knives for cryo-temperatures (Diatome) were used for the trimming (model cryotrim 45°) as well as the cutting process (model cryo 35°).

The antibacterial activity was tested against Gram-positive Staphylococcus aureus (*S. aureus*, ATCC 25923) as well as Gram-negative Escherichia coli (*E. coli*, ATCC 25922) and Pseudomonas aeruginosa (*P. aeruginosa*, ATCC 15692), which are known as the most common bacteria responsible for nosocomial infections [46,47,48]. In order to demonstrate that the PLA@Ag retractor is capable of tolerating the demands of the operating room, the antimicrobial properties were tested after being exposed to steam sterilization in order to determine whether the silver deposited film would resist without being destroyed. The antimicrobial activity of the PLA@Ag retractor was assessed according to the standard shake flask method (ASTM-E2149-01). The method provides quantitative data for measuring the reduction rate in a specific CFU number at a specific time. The CFU is converted further to the average colony-forming units per milliliter (CFU·mL^−1^) of buffer solution in the flask. Initially, *S. aureus*, *P. aeruginosa* and *E. coli* cultures of bacteria were grown on nutrient agar overnight. These cultures were then transferred into a nutrient broth (NB) at an initial optical density (OD) of 0.1 at 660 nm and allowed to grow at 37 °C and 110 rpm with aeration. When the cultures reached an optical density of 0.3 at 660 nm (the beginning of the logarithmic phase), they were centrifuged and washed twice with saline at pH 6.5 to yield a final bacterial concentration of approximately 10^8^ CFU·mL^−1^. Thereafter, 4.5 mL of a saline solution containing a PLA@Ag small piece (~0.5 gr) was poured into a vial with an inner diameter of 2.5 cm, while 500 μL of the strain cells was pipetted into the vial. As such, the initial bacterial concentration in the vial was ~10^7^ CFU·mL^−1^. The three different bacterial suspensions prepared were incubated and then shaken at 37 °C and 230 rpm for up to 120 min (2 hrs). Finally, samples of 100 μL each were taken at a specified time (30, 60 and 120 min), diluted tenfold in saline and then transferred onto nutrient agar plates. The plates were allowed to grow at 37 °C for 24 h to determine the number of surviving bacteria. Antimicrobial activity is reported in terms of percentage of bacteria reduction, calculated as the ratio between the number of surviving bacteria before and after the contact with the control (PLA) and Ag-coated PLA@Ag retractors, using the following formula

(1) Bacteria reduction (%)=((A−B)/A)×100

where *A* and *B* are the average number of bacteria before and after the contact with the Ag-coated PLA@Ag retractor. The experimental protocol was performed three times (*n* = 3) for each bacteria strain, while the antibacterial activity of the PLA@Ag against *S. aureus*, *P. aeruginosa* and *E. coli* after 30, 60 and 120 min of contact is presented as the mean ± standard deviation (SD) value. All the data were analyzed by one-way analysis of variance (ANOVA) and differences between the means were assessed with Neuman–Keuls’s multiple comparison tests to determine the significant variation of the PLA@Ag bactericidal activity with the PLA control sample. Differences were considered significant at *p* < 0.05. 

## 3. Results and Discussion

### 3.1. UV-Vis Spectroscopy of AgNPs Formed by the Sonochemical Reaction

**Figure 2:**
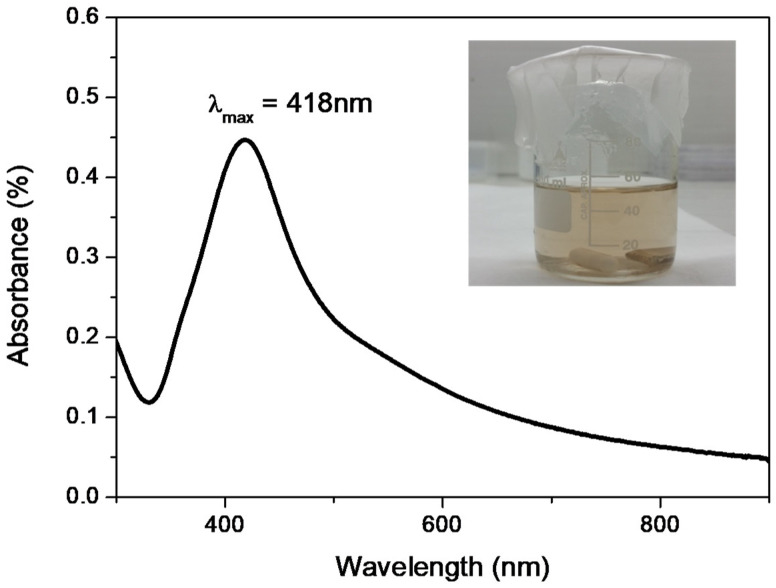
UV-vis spectrum of AgNPs formed by the sonochemical reaction with an LSPR peak centered at ca. 418 nm (inset: photo of free AgNPs after the sonochemical reaction indicating the yellowish color of the solution, characteristic of the AgNP plasmonic effect).

Figure 2 shows the UV-vis spectrum of free AgNPs obtained from the reaction medium after the end of the sonochemical reaction used to fabricate the PLA@Ag retractor. As can be seen in the optical image given as an inset, the color of the solution is slightly yellowish, indicating the existence of nanometer-scale Ag particles. This is more precisely explained by the Ag plasmonic nature when found in nanoscale dimensions, exhibiting the well-known localised surface plasmon resonance (LSPR) effect [49,50]. More specifically, the LSPR peak of AgNPs appears with a maximum centered at ca. 418 nm. The presence of a minimum at ca. 320 nm can be also observed, characteristic of the interband transition in the metal that damps the plasmon oscillation in this spectral region [51]. The UV-vis spectrum, which reveals the formation of AgNPs, is an indirect proof that the Ag layer, which has been deposited onto the PLA surface, consists of Ag nanoparticulate matter.

### 3.2. XRD of PLA@Ag Retractor

**Figure 3:**
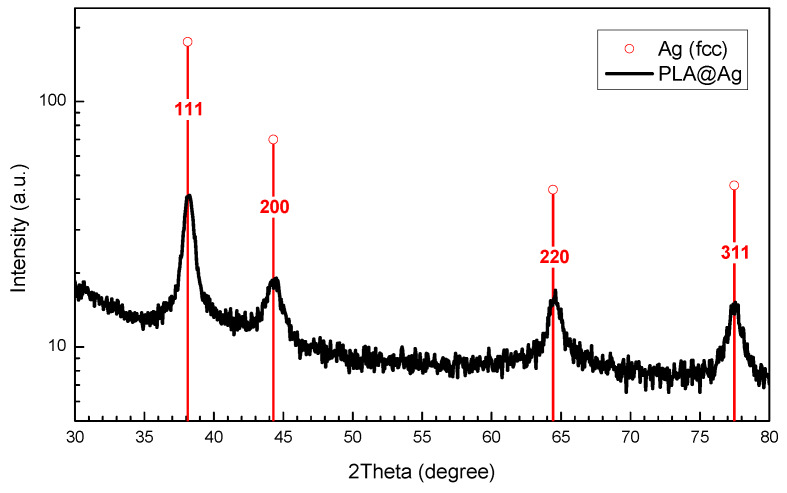
X-ray diffraction patterns of the PLA@Ag retractor.

Figure 3 depicts the XRD pattern of the nanosized Ag-coated PLA@Ag retractor. Sharp diffraction peaks were observed which can be indexed to the face-centered cubic (fcc) structure of metallic Ag (blue lines in Figure 3), with the diffraction peaks corresponding to the (1 1 1), (2 0 0), (2 2 0) and (3 1 1) planes, indicating the formation of pure silver of high crystallinity (JCPDS file, No. 4–783). The crystallite size of the AgNPs was calculated using full width at half maximum (FWHM) of the 100% peak of silver and the Scherrer’s formula

(2) d=Kλ/(β·cosθ)

where *λ*- X-Ray wavelength, *β*-FWHM of the diffraction line, *θ*-diffraction angle, and *K*-constant, generally assumed as 0.9. The calculated average crystallite size of AgNPs was found to be ~25 nm.

### 3.3. TEM Imaging of AgNPs

**Figure 4:**
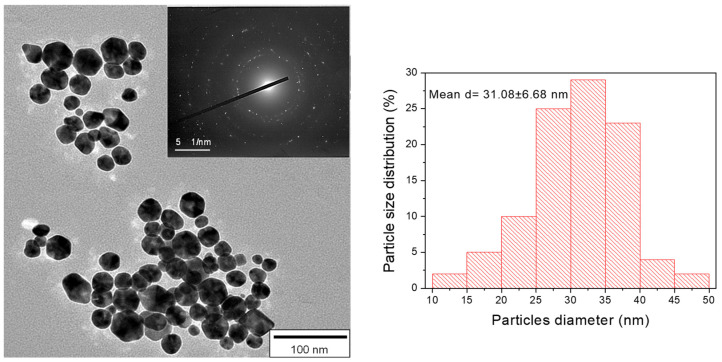
TEM image and the corresponding selected area electron diffraction (SAED) pattern of AgNPs. The histogram of 100 AgNPs measured from the TEM images shows the average particle diameter of 31.08 ± 6.68 nm.

Figure 4 shows the TEM image and the selected area electron diffraction (SAED) pattern of the AgNPs obtained from the reaction medium after the end of the sonochemical reaction. The size of AgNPs was determined by the corresponding TEM images with the illustrated histogram of 100 AgNPs measured showing an average particle diameter of 31.08 ± 6.68 nm. The particle size determined from the TEM analysis is in a good agreement with the XRD calculated AgNP crystallite size.

### 3.4. Surface Morphology of PLA and PLA@Ag

**Figure 5:**
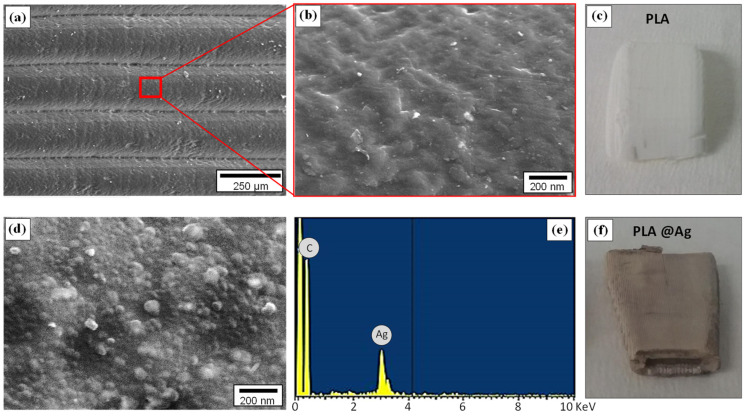
(**a**) SEM images at low magnification showing the 3D printed object layer thickness of approximately 200 μm, and (**b**) at higher magnification, the surface morphology of pure PLA. (**c**) An optical photo of the PLA retractor with white color, (**d**) SEM of PLA@Ag with spherical AgNPs covering the PLA surface and (**e**) the corresponding EDX spectrum. (**f**) An optical photo of the PLA@Ag retractor with a color change to light grey due to the sonochemically deposited AgNPs.

Figure 5 presents the surface morphology of the 3D-printed pure PLA and PLA@Ag retractors. Specifically, Figure 5a shows, at low magnification, the layers of the 3D printed object with a layer thickness of approximately 200 μm, in good agreement with the resolution of the MakerBot 3D printer manufacturer’s technical specifications in terms of printer resolution. The layers observed herein are the main characteristics of the 3D-printing additive-manufacturing technology, which creates a 3D object via the successive addition of layers. The pure PLA retractor shows an amorphous-like morphology, characteristic of polymeric materials (Figure 5b), while in Figure 5c an optical photo of the retractor with a white colour is depicted. In Figure 5d, the surface nanomorphology of the PLA@Ag is shown. As can be observed, a nanograined, homogenous and dense AgNP layer has been successfully formed via the sonochemical deposition, which is responsible for the antimicrobial properties endowed to the surgical instrument intended in this research work. Figure 5e represents the EDX spectrum acquired from the PLA@Ag surface exhibiting the existence of an Ag element, while in Figure 5f the optical photo of the retractor with a light grey color is given.

### 3.5. TEM Imaging of the PLA@Ag Crossection

**Figure 6:**
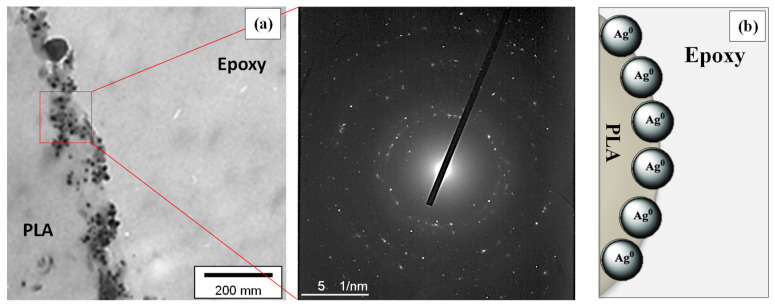
(**a**) TEM image of the PLA@Ag retractor crossection and the corresponding SAED pattern showing the AgNP layer nanoscale characteristics. (**b**) A cartoon illustrating the main finding from the TEM crossection analysis, revealing a “penetration” effect of the AgNPs onto the PLA outer surface (few nanometers) due to the chemical interactions of PLA polymer chains chemistry with AgNPs surface chemistry as well as the sonochemical mechanism followed in this work.

TEM analysis of the PLA@Ag crossection was used in order to study the AgNP nanocoating characteristics, i.e., thickness, as well as realise the quality of the AgNP film, i.e. plausible discontinuities, as a result of the sonochemical growth mechanism followed in this work. The AgNP coating growth could suggest that it can be affected both by the sonochemical experimental parameters as well as the nanoscale chemical interaction of the Ag precursor with the PLA surface macromolecular chains. Figure 6a shows the TEM image of the PLA@Ag retractor ultra-thin film crossection prepared by ultramicrotomy (thickness of ~70 nm). The SAED pattern is also shown revealing highly crystalline AgNPs due to a great number of obtained reflections by the different Ag crystalline plane. The Ag-layer consisting of AgNPs exhibits a poly-crystalline nature typical for nanoparticle consisting thin films. As can be observed, the AgNPs are well distributed along the PLA surface with a dense and homogeneous nanoparticle layer formed with a thickness ~80 nm. Moreover, AgNPs are found to have also penetrated to the bulk of PLA, possibly allowing a good adhering AgNP layer with the PLA retractor surface. This will allow the efficient Ag ion release against biofilm formation without AgNP detachment during use. TEM crossection analysis proves that the sonochemical method utillised herein for the wet-chemical deposition of a AgNP layer onto PLA 3D object surface is very promising due to the versatility as well as the possibility to stabilize the NPs onto the polymer substrate material. A plausible mechanism is that the PLA polymeric chains are partially swollen during sonication in the reaction medium, thus allowing the Ag metal source ions to penetrate into the polymer chains, with some nanometers getting entrapped and further reduced during sonochemistry. All the chemical interactions and AgNP layer growth mechanisms have been explained in detail above in Section 2 as well as shown in Figure 1.

Adhesion or nanoindentation tests should be performed for the PLA@Ag retractors in order to get a direct and quantitative proof for the adhesion strength of the AgNP layer with the underlying PLA substrate material, since TEM shows only the morphological and microstructure characteristics. This can further support the potential for practical application of the PLA@Ag retractors in the future as antimicrobial surgical equipment.

### 3.6. Bactericidal Tests

**Figure 7:**
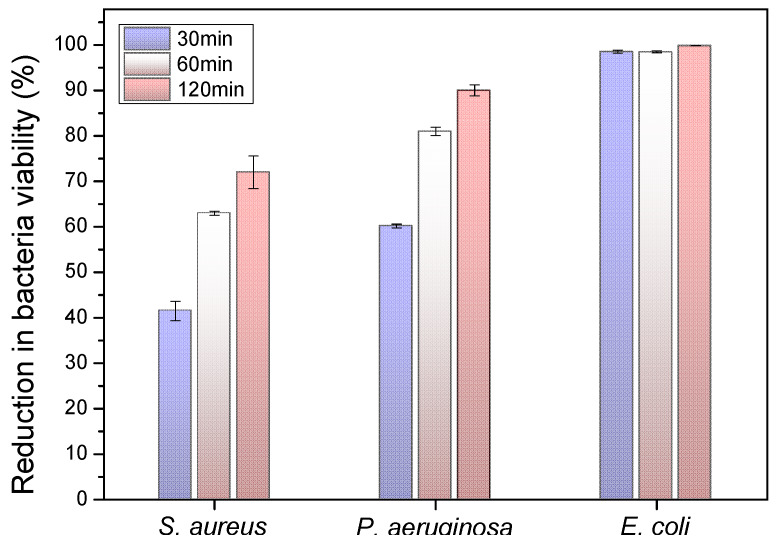
Antibacterial activity of PLA@Ag towards *E. coli*, *P. aeruginosa* and *S. aureus* after 30, 60 and 120 min of contact. Values are mean ± SD (error bars) for three tests (details in the experimental part). Mean values do not differ significantly (*p* < 0.05).

The antibacterial activity of the PLA@Ag retractor was examined by a reduction in *S. aureus*, *P. aeruginosa* and *E. coli* bacteria viability (%) at 30, 60 and 120 min duration of contact. Differences were considered significant at *p* < 0.05. It can be seen in Figure 7 that all cultures of the bacteria were eradicated completely after 120 min of treatment (*p* < 0.05). The detailed results are presented in Table 1 (values shown represent the means ± SD of triplicate measurements; *n* = 3). It can be observed that, even in 60 min of *P. aeruginosa* and *E. coli* treatment with PLA@Ag, more than 80% of the bacteria have been eradicated. The observed required time window of 120 min to reduce the initial *S. aureus*, *P. aeruginosa* and *E. coli* amount of CFU to zero is governed by the velocity of Ag^+^ ion release, which is responsible for the antibacterial activity (*p* < 0.05). It is worth mentioning that the “ideal” case scenario for the main bactericidal mechanism would be only the release of silver ions from the AgNPs attached onto the PLA surface. The Ag nanolayer created in our work is expected to be highly stable and strongly adherant to the PLA surface due to the in-situ growth and attachment of AgNPs during the sonochemical synthesis. However, it is highly possible that some AgNPs not strongly bound with the PLA surface could be detached after the bacteria contamination/attachment and/or during the bacteria biofilm formation. It is worth mentioning that PLA@Ag retractors have been cleaned with copious amounts of water to remove any weakly bound AgNPs, as well as sterilised before all performed antibacterial tests. Any possibly detached AgNPs from the PLA surface could have a further bactericidal effect, since AgNPs are known to anchor to the bacterial cell wall and consequently infiltrate it so that Ag+ will be released and kill further the bacteria. However, this is not the ideal scenario and should be avoided from bactericidal surfaces, since the AgNP-consisting coating will not be stable over time and will lose its bactericidal properties over time. From the SEM and TEM crossection microscopy analyses, it can be deduced that the AgNP layer seems to be well attached to the PLA surface, with some AgNPs penetrating a few nanometers in the bulk of PLA endowing a “fixation” mechanism of the coating with the substrate. However, they have to be permed in future adhesion and nanoindentation tests to study quantitatively the AgNP nanocoating adhesion strength with the PLA surface, so as to realise the potential practical application of the PLA@Ag antimicrobial retractor devices in daily use as well as transferring this technology to a wide range of other surgical equipment. Overall, the sonochemically prepared PLA@Ag retractor exhibits excellent antibacterial properties with the greatest bactericidal effect on *E.coli* (98.48%) and the lowest on *S. aureus* (63.05%) at 60 min (*p* < 0.05). The differences found in bacteria viability between Gram-positive and Gram-negative strains is in good agreement with findings from a recent study by Huq et al. [52]. Specifically, in this work, the growth curves of *P. aeruginosa* and *S. aureus* cultured in R2A broth with various concentrations of AgNPs have been shown, demonstrating a higher AgNPs susceptibility of Gram-negative bacteria compared to that of Gram-positive bacteria. This has been more precisely attributed due to distinctions in the composition of the bacteria cell wall, which have been reported elsewhere [53]. The PLA surfaces of bare PLA retractor exhibited no bactericidal effect.

## 4. Conclusions

PLA@Ag Army/Navy retractors with antimicrobial activity and on demand geometry have been fabricated with a fast, simple, versatile and easy two-step protocol based on 3D printing and the sonochemical deposition of thin films. Typical Army/Navy retractors have been selected as a use case of surgical instrument since they can be easily infected during operations. It is estimated that the antimicrobial retractors fabricated herein could replace the stainless steel counterpart with at least ten times lower cost, considering that each instrument required only 0.34€ of PLA while the cost of chemicals was less than 1€ for the 250 mL reaction medium. Due to the unprecedented accessibility of 3D printing technology world-wide, as well as the cost efficiency of these antimicrobial instruments, there are numerous implications for surgery equipment with a possible daily application in underserved and less developed parts of the world as well as in military and aerospace missions. It is envisaged that the described protocol will open a new era in the rapid prototyping of surgical equipment and medical devices.

## Figures and Tables

**Table 1:** Antibacterial Activity of the PLA@Ag against *S. aureus*, *P. aeruginosa* and *E. coli* after Different Incubation Time (*n* = 3, *p* < 0.05).

Reduction in Viability, %			
	30 min	60 min	120 min
*S. aureus*	41.50 ± 2.12	63.05 ± 0.45	72.15 ± 3.58
*P. aeruginosa*	60.17 ± 0.42	81.12 ± 0.95	90.18 ± 1.22
*E. coli*	98.54 ± 0.32	98.48 ± 0.23	99.86 ± 0.04
